# Is bone mineral density in middle-aged and elderly individuals associated with their dietary patterns? A study based on NHANES

**DOI:** 10.3389/fnut.2024.1396007

**Published:** 2024-08-23

**Authors:** Huang Runting, Luo Qingyue, Yuan Yining, Shu Huiyu, Yang Shu, Feng Xixi

**Affiliations:** ^1^Department of Public Health, Chengdu Medical College, Chengdu, China; ^2^School of Medical Information Engineering, Chengdu University of Traditional Chinese Medicine, Chengdu, China

**Keywords:** dietary pattern, bone mineral density, factor analysis, cluster analysis, cross-sectional study

## Abstract

**Introduction:**

Bone mineral density (BMD) is a crucial index for predicting fracture risk and diagnosing osteoporosis. With the global rise in osteoporosis prevalence, understanding the relationship between dietary patterns and BMD is vital for public health. This study aimed to explore the association between various dietary patterns and BMD among adults using data from the National Health and Nutrition Examination Survey (NHANES).

**Methods:**

Data were analyzed from 8,416 NHANES participants aged 40 years and older across three non-consecutive survey cycles from 2013 to 2020. Dietary patterns were identified using a combination of factor analysis and cluster analysis. BMD measurements were then assessed, and associations with the identified dietary patterns were analyzed, with adjustments made for demographic variables.

**Results:**

The analysis identified three distinct dietary patterns: “Low protein-High Dietary fiber-Vitamin A-Magnesium (LP-HDF-Vit A-Mg)”, “High macronutrient-Choline-Selenium (HM-Cho-Se)”, and “Low macronutrient-Vitamin D-Calcium (LM-Vit D-Ca)”, and then we found that women, older adults, and certain ethnic groups were at higher risk for low BMD. Participants adhering to the “HM-Cho-Se” and “LP-HDF-Vit A-Mg” dietary patterns exhibited significantly higher BMD compared to those following the “LM-Vit D-Ca” pattern. After adjusting for demographic variables, the “HM-Cho-Se” pattern remained positively associated with BMD, while the “LM-Vit D-Ca” pattern showed no significant association with BMD or the risk of low BMD.

**Discussion:**

The findings suggest that adherence to the “HM-Cho-Se” dietary pattern may reduce the risk of low BMD, indicating potential synergies between these nutrients for bone health. However, the study has limitations, including the cross-sectional design and potential subjectivity in factor analysis. Future research should focus on longitudinal studies involving diverse age groups to better understand the causal relationship between dietary patterns and BMD. Despite these limitations, the study highlights the importance of dietary factors in maintaining bone health and suggests potential dietary interventions to reduce the risk of low BMD and osteoporosis.

## 1 Introduction

Bone mineral density (BMD) is a crucial indicator of bone mass per unit volume or area, serving as a significant predictor of fracture risk, an essential diagnostic parameter for osteoporosis and osteopenia, and a prevalent metric for evaluating bone health (1). It is of great concern that individuals with low BMD face a significantly elevated risk of developing osteoporosis and sustaining fractures. Osteoporosis results in a reduction in bone strength and resilience, rendering even minor traumas sufficient to cause fractures, particularly in the hip, spine, and wrist. Among these, hip fractures are of particular concern, as they often necessitate surgical intervention and carry a high mortality rate (2). A significant increase in mortality is observed in elderly patients within the first year after a hip fracture, largely due to complications from surgery and prolonged bed rest, such as infections, blood clots, and cardiorespiratory failure (3).

In the 21st century, osteoporosis due to low BMD has emerged as one of the top five global diseases, with a prevalence of 23.1% in women and 11.7% in m en. This condition has become a significant public health concern, imposing substantial economic burdens on many countries (4). In the United States alone, approximately 2 million fractures are attributed to osteoporosis annually, with projected healthcare costs reaching $25.3 billion by 2025 (5). Furthermore, there is evidence that BMD among United States adults has been declining over the past decade, which may have a detrimental impact on the situation (6).

The development of BMD is influenced by a number of factors, including biological genetics, metabolic processes, and habits (7). It is noteworthy that dietary nutritional intake plays a pivotal role in the presence and development of low BMD and is a factor that can be readily modified (8). The research evidence is clear that an adequate intake of essential nutrients, including protein, calcium, vitamin D, vitamin K, magnesium, and phosphorus, is essential for maintaining bone health and reducing the risk of low BMD (9). This underscores the potential of dietary modifications to mitigate bone loss.

As societies progress, dietary patterns become increasingly intricate. Traditional research often isolates the effects of individual nutrients or foods on diseases, but such approaches fail to capture the interactions among various nutrients. Consequently, dietary pattern analysis has emerged as a supplementary methodology for investigating the association between diet and the risk of developing chronic diseases (10). Previous studies have identified representative dietary patterns, including the Mediterranean diet, which is rich in meat, eggs, milk, nuts, and olive oil, and the Nordic diet, which is characterized by whole grains, root vegetables, and berries (11–13). Despite the 2015 United States Dietary Guidelines Advisory Committee’s emphasis on considering nutrient amounts, proportions, types, combinations, and consumption frequency in dietary patterns, there remains a dearth of nutrient-based studies examining the relationship between dietary patterns and disease (11–14). Although food patterns can predict certain disease risks, the specific mechanisms remain unclear; however, the combination of nutrients may exert a more significant effect on disease than individual nutrients, suggesting that studying dietary patterns could offer deeper insights into the underlying biological mechanisms and new perspectives on disease etiology, while nutrient-based analyses allow for comparisons of nutritional status across diverse populations irrespective of behavioral, cultural, or geographical differences (15–17). A number of studies have employed nutrients as a means of investigating the relationship between dietary patterns and disease, including obesity, psychological disorders, metabolic syndrome, and certain cancers (15, 18–20). However, fewer studies have been conducted on the association between dietary patterns and bone health (21).

A review of the literature revealed that factor analysis and cluster analysis are the most commonly used methods in dietary pattern analysis. Factor analysis yields factor scores, which are subsequently categorized into quartiles to depict dietary patterns. However, this method may result in a loss of statistical information (22). Cluster analysis, which encompasses Q-type (sample clustering) and R-type (variable clustering), offers distinct advantages. Q-type clustering categorizes dietary variables but fails to elucidate intra-group dietary patterns. In contrast, R-type clustering excels at distinguishing populations, although the complexity and icorrelation of dietary items challenge the interpretability of the results (23).

In this study, we will integrate factor analysis and cluster analysis methods to explore the relationship between dietary patterns based on nutrient intake and BMD. Initially, factor analysis will extract factor scores representing nutrient intake from participants, thereby identifying distinct dietary patterns. Subsequently, cluster analysis will effectively classify the study population, maximizing the retention of raw dietary information and distinguishing populations with different dietary patterns. Finally, we will analyze the correlations between these dietary patterns and participants’ BMD, specifically examining how nutrient combinations collectively affect BMD. Our findings aim to inform the development of nutrient-based dietary interventions to prevent and minimize low BMD and osteoporosis. Through our innovative approach and comprehensive analysis, we hope to contribute new perspectives and methodological advancements to the field of diet and BMD research, providing empirical support for future studies.

## 2 Materials and methods

### 2.1 Data source

The National Health and Nutrition Examination Survey (NHANES) is a research program designed to assess the health and nutritional status of adults and children through interviews and physical examinations. It is administered by the National Center for Health Statistics (NCHS), a division of the United States Centers for Disease Control and Prevention. Beginning in the 1960s, it was transformed in 1999 into a continuous program focusing on different groups and health topics. Approximately 5,000 people from 15 counties are surveyed each year. Interviews include demographics, socioeconomics, diet, and health, while screenings include medical, dental, and physiologic measures, as well as laboratory tests. NHANES is designed to determine disease prevalence and risk factors and to assess nutritional status. These data inform epidemiologic studies, health research, and public health policy. The database is available at www.cdc.gov/nchs/NHANES, the official website of NHANES.

### 2.2 Study population

**Figure 1:**
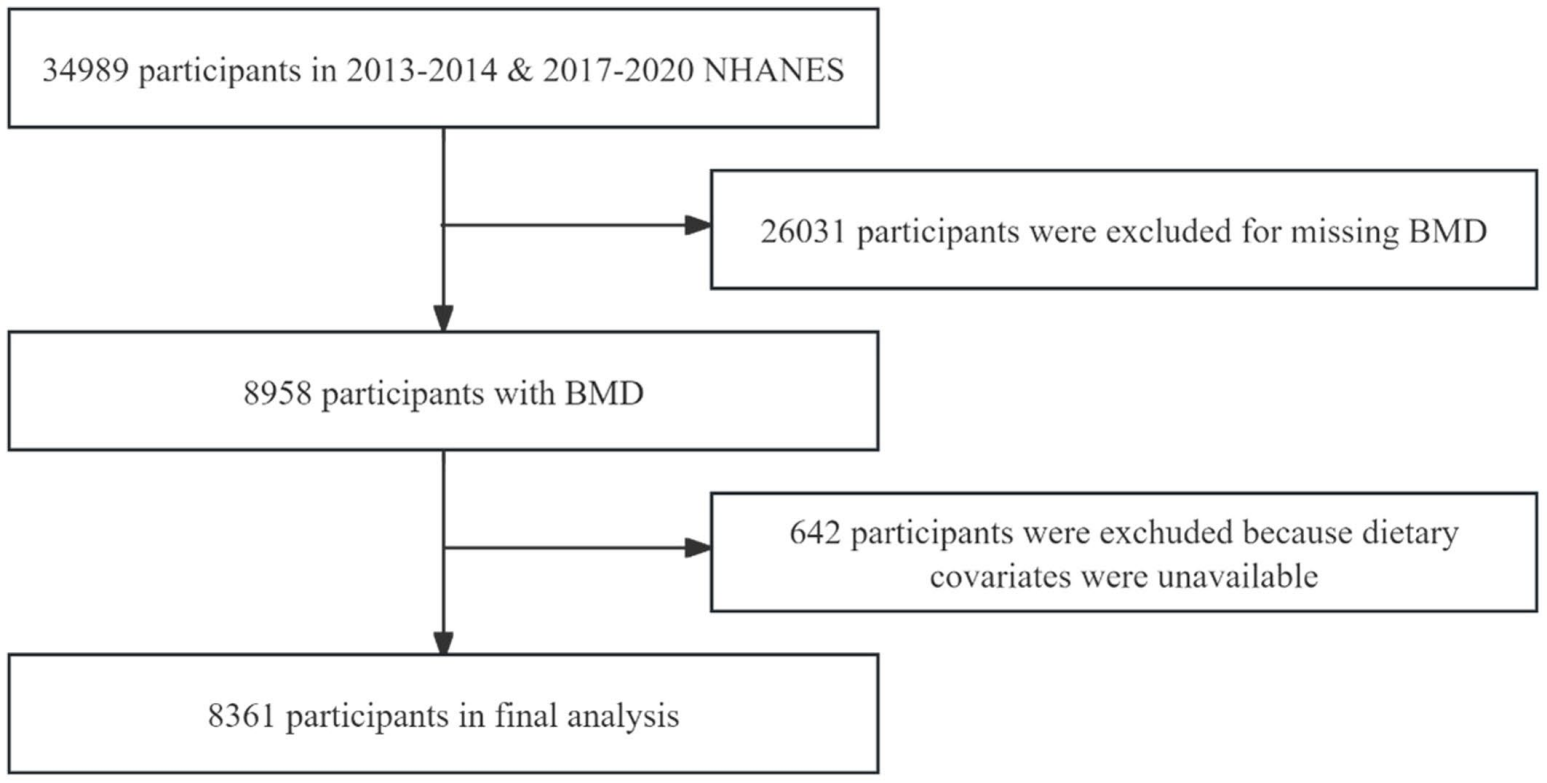
Flowchart of the sample selection.

In our study, data from three survey cycles from 2013 to 2014, 2017–2020 were used and the three survey cycles included 34,989 participants. After excluding participants under 40 years of age, those who had used contrast agents in the past 7 days, pregnant individuals, those weighing over 204 kilograms or measuring over 2.17 meters in height, and participants with missing scans of the femur region tissue (*n* = 26,031), as well as those with missing dietary recall information (*n* = 642), the final analyzed sample consisted of 8,316 individuals. The NHANES protocol was reviewed and approved by the NCHS Research Ethics Review Board (24). The sample screening process is shown in Figure 1.

Data collection for the NHANES 2019–2020 cycle was not completed due to the 2019 coronavirus disease (COVID-19) pandemic. Therefore, data collected in March 2019–2020 were combined with selected data from the NHANES 2017–2018 cycle to form a nationally representative sample of NHANES March 2017–2020 pre-pandemic data.

### 2.3 Outcome variable: femur neck skeletal status

In this study, bone mineral density (BMD) was measured in grams per square centimeter (g/cm^2^) using dual-energy X-ray absorptiometry (DXA). The DXA scanning was performed with the HOLOGIC QDR 4500A fan beam densitometer, specifically focusing on the femoral neck. The which is calculated by comparing a patient’s BMD to the BMD of healthy young adults of the same gender, was determined using the following formula: $T_{-}\mathrm{score}=\frac{BMD_{\mathrm{research}}-BMD_{\mathrm{reference}}}{SD_{\mathrm{reference}}}$ (25). In accordance with the World Health Organization (WHO) recommendations, the mean and standard deviation of femoral neck BMD in healthy adults were used as references. These reference values were based on data from white women aged 20–29 years, as reported in the NHANES 2005–2008 study (26). BMD values were classified according to the T-score classification standard recommended by the WHO and the osteoporosis questionnaire. For the detailed definition and classification, refer to Table 1.

**Table 1:** Classification of BMD based on T-score and osteoporosis questionnaire.

Source	Levels	Definition	Outcome variable
Calculated T-score	T-score ≥ -1SD	Normal bone mass	Normal BMD
	-2.5SD<T-score<-1SD	Low bone mass	Low BMD
	T-score ≤ −2.5SD	Osteoporosis	Low BMD
Osteoporosis questionnaire	–	Self-reported osteoporosis^*^	Low BMD

*Respondents will be asked to answer the question “Has a doctor or health professional ever told you that you have osteoporosis? Those who answered “yes” were defined as self-reported osteoporosis patients.

### 2.4 Exposure variable: dietary patterns

Dietary data were obtained from the “What We Eat in America (WWEIA)” section of NHANES. Daily nutrient intakes were averaged from two 24-h dietary surveys. The nutrients studied included protein, carbohydrate, dietary fiber, saturated and unsaturated fatty acids (SFA&UFA), cholesterol, vitamins A, D, E, K, C, B, choline, calcium, phosphorus, magnesium, iron, zinc, copper, sodium, potassium, and selenium, totaling 22 nutrient groups. Detailed nutrient deletions and combinations are shown in Table 2.

**Table 2:** Nutrients are calculated by the FNDDS groups and modified groups used in the present study.

The original nutrients of NHANES	The modified nutrients
1. Protein(g)	1.Protein(g)
2. Carbohydrate(g)	2.Carbohydrate(g)
3. Total Sugars(g)	Removed
4. Dietary Fiber(g)	3.Dietary Fiber(g)
5. Total Fat(g)	Removed
6. Total Saturated Fatty Acids(g)	4.Saturated Fatty Acids, SFA(g)
7. Total Monounsaturated Fatty Acids(g)	5.Unsaturated Fatty Acid, UFA(g)
8. Total Polyunsaturated Fatty Acids(g)	
9. Cholesterol(mg)	6.Cholesterol(mg)
10. Vitamin E as Alpha-Tocopherol(mg)	7.Vitamin E(mg)
11. Added Alpha-Tocopherol (Vitamin E)(mg)	
12. Retinol(ug)	8.A-Vitamins(mg)
13. Vitamin A, Rae(ug)	
14. Alpha-Carotene(ug)	
15. Beta-Carotene(ug)	
16. Beta-Cryptoxanthin(ug)	
17. Lycopene(ug)	
18. Lutein + Zeaxanthin(ug)	
19. Thiamin (Vitamin B1) (mg)	9.B-Vitamins(mg)
20. Riboflavin (Vitamin B2) (mg)	
21. Niacin(mg)	
22. Vitamin B6(mg)	
23. Vitamin B12(ug)	
24. Added Vitamin B12(ug)	
25. Total Folate(ug)	
26. Folic Acid(ug)	Removed
27. Food Folate(ug)	
28. Folate, DFE(ug)	
29. Total Choline(mg)	10.Choline(mg)
30. Vitamin C(mg)	11.Vitamin C(mg)
31. Vitamin D (D2 + D3) (ug)	12.Vitamin D(ug)
32. Vitamin K(ug)	13.Vitamin K(ug)
33. Calcium(mg)	14.Calcium(mg)
34. Phosphorus(mg)	15.Phosphorus(mg)
35. Magnesium(mg)	16.Magnesium(mg)
36. Iron(mg)	17.Iron(mg)
37. Zinc(mg)	18.Zinc(mg)
38. Copper(mg)	19.Copper(mg)
39. Sodium(mg)	20.Sodium(mg)
40. Potassium(mg)	21.Potassium(mg)
41. Selenium(ug)	22.Selenium(ug)

We are going to summarize the dietary patterns of nutrient extraction from the 22 nutrient groups. Dietary patterns were extracted using factor analysis combined with cluster analysis, and the specific analysis steps were as follows (1): In order to overcome the effect of outliers, nutrient intake was standardized for all the nutrient variables after 5% bilateral tailing (27). (2) Factor analysis requires the existence of correlation between the original variables, therefore, Kaiser-Mayer-Olkin (KMO) test and Bartlett’s test of sphericity were used to analyze the correlation between the nutrient variables and determine whether the data were suitable for factor analysis. (3) K-means cluster analysis was used to analyze the factor scores (FS) of all samples for each type of nutrient intake.

### 2.5 Other covariates

The covariates included in the study were age, sex, race, marital status, education, family income, Body Mass Index (BMI), physical activity, smoking history, and alcohol consumption history. According to the Family poverty income ratio (PIR), family income are divided into three levels. Physical activity was assessed by weekly metabolic equivalent task (MET) minute aggregated scores, according to the NHANES recommendations. Weekly MET-minutes were calculated as follows: [8.0 MET scores (weekly minutes of vigorous work-related activity + weekly minutes of vigorous leisure-time physical activity)] + [4.0 MET scores (weekly minutes of moderate work-related activity + weekly minutes of moderate leisure-time physical activity + weekly minutes Walking or bicycling for transportation)] (28). Detailed levels are shown in Table 3.

**Table 3:** Other covariates and their grouping details.

Variables	Levels	Grouping basis
Age	①40 ~ 55y; ②55 ~ 70; ③ ≥ 70	–
Sex	①Male; ②Female	–
Race	①Mexican Americans;②Non-Hispanic White people;③Non-Hispanic Black people; ④Non-Hispanic Asians;⑤Other Hispanics;⑥Other Races	–
Marital status	①Never married;②Married/Living with partner;③Widowed/Divorced/Separated	–
Education	①Below high school;②High school; ③Above high school	–
Family income	①low income;②middle income;③high income;	Percentage of poverty guidelines for eligibility determined by the federal program (29): ①PIR < 1.85;②1.85 ≤ PIR ≤ 3.5;③PIR >3.5
BMI	①BMI<18.5;②18.5 ≤ BMI ≤ 24.9; ③25 ≤ BMI ≤ 29.9;④ BMI ≥ 30	WHO definition of obesity (30)
Physical activity	①insufficiently active;②moderately active;③highly active	NHANES recommendations (28): ①Weekly MET-minutes <600; ②600 ≤ Weekly MET-minutes ≤3,000; ③Weekly MET-minutes >3,000
Smoke history	①no;②yes	–

## 3 Statistical analyses

Sample characteristics were described using mean and standard deviation (SD) for continuous variables and N (%) for categorical variables. The chi-square test or analysis of variance (ANOVA) was used to test the differences between groups for categorical or continuous variables. To investigate the correlation between femoral neck BMD and dietary patterns, multivariate linear regression and logistic regression were performed. Multivariate models were adjusted for demographic variables such as age, sex, and race. NHANES uses complex sampling designs, including multistage whole-cluster sampling and weighting methods, to oversample individuals in certain groups to ensure adequate statistical power. We constructed weights for the multi-period combined data by selecting sampling weights associated with the minimum subsample (MEC), as recommended by NHANES (31). All coefficients with 95% confidence intervals (CI) were recorded. *p* values less than 0.05 (two-sided) were considered statistically significant. All statistical analyses were performed using the R software (version 4.3.1).

## 4 Outcome

### 4.1 Characteristics of participants

The characteristics of the study population were presented in Table 4. A total of 8,316 participants were included in the study, comprising 4,489 individuals with normal bone mass and 3,827 with low BMD. The minimum age of the study participants included in this investigation was 40 years old, with a maximum age of 80 years old, primarily concentrated in the age group of 55 to 70 years old. Among them, 4,299 were males (51.7%) and 4,017 were females (48.3%). See Table 4 for details.

**Table 4:** General demographic characteristics.

Variables	Levels	Normal BMD	Low BMD	χ^2^/*t*	*p*
*n*		4,489	3,827		
Age group (%)	40 ~ 55	1,412 (31.5)	606 (15.8)	480.70	<0.001
	55 ~ 70	2,280 (50.8)	1833 (47.9)		
	≥70	797 (17.8)	1,388 (36.3)		
Gender (%)	Male	2,867 (63.9)	1,432 (37.4)	577.66	<0.001
	Female	1,622 (36.1)	2,395 (62.6)		
Ethnicity (%)	Mexican Americans	552 (12.3)	360 (9.4)	395.98	<0.001
	Non-Hispanic White people	1,564 (34.8)	1865 (48.7)		
	Non-Hispanic Black people	1,374 (30.6)	565 (14.8)		
	Non-Hispanic Asians	378 (8.4)	539 (14.1)		
	Other Hispanics	477 (10.6)	383 (10.0)		
	Other Races	144 (3.2)	115 (3.0)		
Education (%)	Below high school	920 (20.5)	750 (19.6)	1.77	0.413
	High school	1,065 (23.8)	948 (24.8)		
	Above high school	2,497(55.7)	2,124 (55.6)		
Marital (%)	Never married	562 (14.0)	499 (14.1)	118.84	<0.001
	Married/Living with partner	2,804 (69.9)	2,113 (59.9)		
	Widowed/Divorced/Separated	644 (16.1)	918 (26.0)		
Family income (%)	Low income	1,518 (38.3)	1,451 (41.9)	16.43	<0.001
	Middle income	968 (24.4)	873 (25.2)		
	High income	1,474 (37.2)	1,137 (32.9)		
Weight (%)	Underweight	12 (0.3)	78 (2.0)	534.10	<0.0011
	Normal	732 (16.4)	1,284 (33.7)		
	Overweight	1,630 (36.4)	1,427 (37.4)		
	Obesity	2098 (46.9)	1,023 (26.8)		
Physical activity (%)	Insufficiently active	1798 (40.1)	1790 (46.8)	68.30	<0.0011
	Moderately active	1,382 (30.8)	1,211 (31.6)		
	Highly active	1,309 (29.2)	826 (21.6)		
Smoke (%)	No	2,290 (51.0)	2,140 (55.9)	19.77	<0.0011
	Yes	2,199 (49.0)	1,687 (44.1)		

### 4.2 Dietary pattern

**Figure 2:**
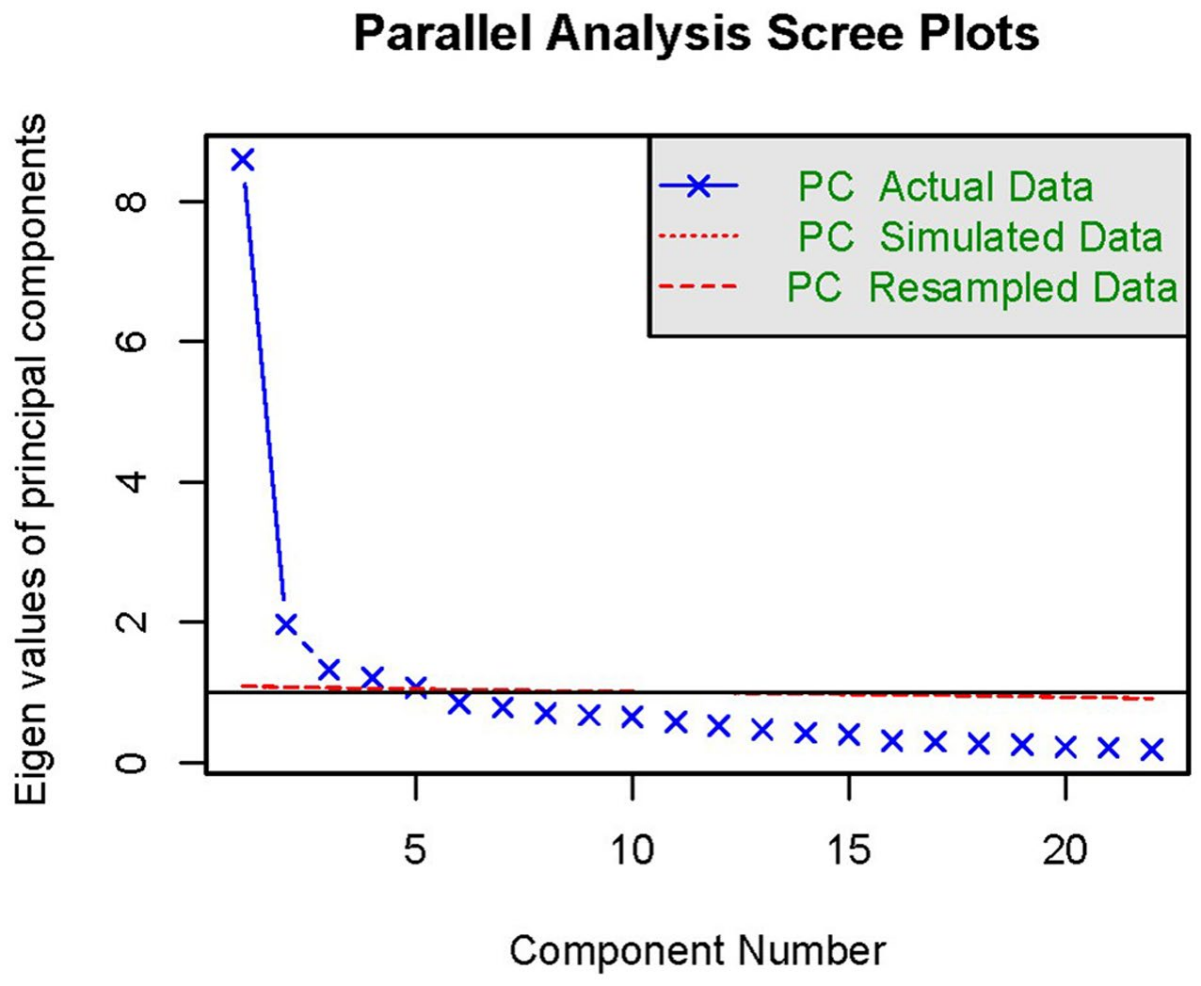
Parallel analysis scree plots.

According to the statistical results, the sample *KWO* test value was 0.92 and *Barlen’s Chi-Squar* was 96844.06, with a probability of concordance <0.001, indicating strong correlation between the nutrients, which is suitable for factor analysis. The results of factor analysis showed that five factors had an eigen root >1 (Figure 2). Three dietary factors were identified with a total variance contribution ratio of 54.12% by considering the eigen root, total variance contribution ratio, number of factors before the inflection point in the fragmentation plot, and the appropriateness of nutrient combinations. In order to obtain more typical factor compositions and make the results easier to interpret, the original factor loading matrices were rotated with variance-maximizing orthogonal rotation of the rotated loading matrices. Nutrients with factor loadings >|0.40| were used as the main contributing terms of the factors, and the different nutrients contained in each factor were categorized for the naming of dietary patterns.

Factor load after rotation was shown in Table 5. The first factor was dominated by nutrients such as protein, lipids, choline, phosphorus, zinc, sodium, potassium and selenium. The second factor was dominated by nutrients such as carbohydrates, dietary fiber, vitamins A, E, K, C, magnesium, iron, and potassium, and the third factor was dominated by vitamin D, calcium, iron, zinc, and copper.

**Table 5:** Factor loadings after orthogonal rotation with maximum variance.

Nutrients	Factor 1	Factor 2	Factor 3
Protein	**0.767**	0.222	0.248
Carbohydrate	**0.407**	**0.463**	0.131
Dietary fiber	0.176	**0.785**	0.192
Saturated fatty acids	**0.706**	0.134	0.193
Unsaturated fatty acids	**0.690**	0.268	0.060
Cholesterol	**0.718**	−0.131	0.075
A-vitamins	0.057	**0.599**	−0.006
Vitamin D	0.191	−0.068	**0.753**
Vitamin E	0.241	**0.504**	0.386
Vitamin K	0.122	**0.538**	−0.002
Vitamin C	−0.050	**0.564**	0.192
B-vitamins	**0.417**	0.116	**0.765**
Choline	**0.738**	0.087	0.203
Calcium	0.374	0.269	**0.425**
Phosphorus	**0.698**	0.315	0.283
Magnesium	0.391	**0.612**	0.200
Iron	0.377	**0.465**	**0.520**
Zinc	**0.522**	0.326	**0.558**
Copper	−0.082	0.259	**0.492**
Sodium	**0.700**	0.299	0.016
Potassium	**0.441**	**0.580**	0.212
Selenium	**0.738**	0.146	0.191

Bold means factor loadings >|0.40|.

**Figure 3:**
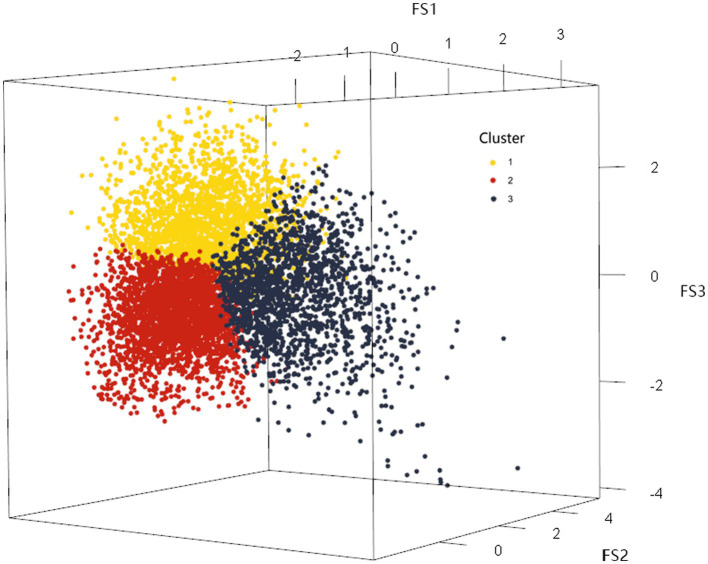
The 3D scatter plot of clustering results based on factor scores.

On this basis, the *K-means* method was used to cluster the factor scores of the first three common factors of the 22 categories of nutrients of the 8,316 research subjects, and the maximum number of iterations was set to the maximum value of 20, and the clustering results were shown in Table 6, and the number of clusters was determined to be 3 according to the results of the factor analysis. Clustering through the factor scores basically divided the 22 categories of nutrients into three types. The division of three types of sample space was shown in the Figure 3. The mean squares of the clusters were analyzed by ANOVA with *p* < 0.001, indicating that the differences in the classification of the three dietary patterns were statistically significant.

**Table 6:** K-means clustering results based on factor scores.

Cluster	FS1	FS2	FS3	SS_within_	SS_tot _between_
Cluster 1	1.02	−0.33	0.21	4869.05	9893.96
Cluster 2	−0.67	−0.55	−0.40	3656.66	
Cluster 3	−0.25	1.19	0.32	6525.33	

Based on the results of cluster analysis and the mean intake of each nutrient within the identified patterns, distinct dietary profiles emerged. Cluster 1 exhibited higher mean intake levels of protein, fat, total choline, phosphorus, zinc, sodium, potassium, and selenium compared to the other clusters. This pattern aligned with the characteristics of dietary Factor 1, characterized by high levels of macronutrients, choline, and selenium. Thus, it was designated as the “High Macronutrient-Choline-Selenium (HM-Cho-Se)” pattern.

Cluster 2 demonstrated lower intakes of protein, carbohydrates, and lipids relative to the other clusters, while exhibiting elevated levels of vitamin D and calcium intake. This dietary pattern mirrored the loading characteristics of Dietary Factor 3, characterized by low macronutrient intake but high levels of vitamin D and calcium. Accordingly, it was labeled the “Low Macronutrient-Vitamin D-Calcium (LM-VitD-Ca)” pattern.

In contrast, Cluster 3 displayed lower protein intake but higher consumption of carbohydrates, dietary fiber, and vitamins A, E, K, and C compared to the other clusters. This intake profile corresponded to the loading characteristics of Dietary Factor 2, featuring low protein intake but high intake of dietary components and vitamins. Therefore, it was denoted as the “Low protein-High Dietary fiber-Vitamin A- Magnesium (LP-HDF-VitA-Mg)” pattern (Table 7).

**Table 7:** Means of nutrient intake for each category of the three clustering patterns [mean (SD)].

Nutrients	Cluster1	Cluster 2	Cluster 3	*p*
Energy(kcal)	2400.71 (808.39)	1781.80 (1019.69)	1963.73 (843.81)	<0.001
Protein	93.93 (29.25)	64.39 (34.52)	60.46 (28.49)	<0.001
Carbohydrate	240.68 (101.49)	185.98 (100.73)	248.02 (99.27)	<0.001
Dietary fiber	15.88 (7.42)	14.20 (8.77)	19.36 (10.00)	<0.001
Saturated fatty acids	31.14 (12.82)	21.30 (12.56)	20.47 (11.66)	<0.001
Unsaturated fatty acid	55.88 (21.69)	37.67 (21.95)	40.82 (22.11)	<0.001
Cholesterol	399.45 (199.44)	234.92 (186.04)	186.98 (147.36)	<0.001
A-vitamins	7.79 (7.17)	7.56 (8.12)	10.98 (10.57)	<0.001
Vitamin D	3.69 (3.55)	5.46 (4.99)	3.59 (4.23)	<0.001
Vitamin E	9.16 (4.89)	8.14 (6.41)	9.71 (6.25)	<0.001
Vitamin K	97.04 (74.48)	88.49 (86.96)	120.94 (116.81)	<0.001
Vitamin C	57.49 (56.41)	65.88 (64.10)	92.46 (76.94)	<0.001
B-vitamins	42.52 (13.93)	33.07 (18.13)	33.12 (16.11)	<0.001
Choline	397.77 (154.20)	273.40 (168.51)	255.37 (138.73)	<0.001
Calcium	809.99 (423.58)	870.51 (493.50)	714.01 (422.68)	<0.001
Phosphorus	1443.37 (489.44)	1128.13 (608.58)	1084.19 (538.81)	<0.001
Magnesium	290.00 (105.93)	251.94 (134.37)	292.63 (144.06)	<0.001
Iron	14.32 (5.46)	11.88 (6.95)	13.47 (6.67)	<0.001
Zinc	11.96 (4.63)	8.91 (5.18)	9.09 (4.95)	<0.001
Copper	1.35 (1.86)	1.55 (3.32)	1.97 (3.79)	<0.001
Sodium	3802.35 (1344.25)	2611.03 (1419.41)	2793.90 (1453.32)	<0.001
Potassium	2580.04 (940.75)	2226.90 (1175.30)	2518.96 (1201.54)	<0.001
Selenium	132.83 (44.88)	90.07 (51.22)	85.36 (45.51)	<0.001

### 4.3 Dietary pattern and BMD of femoral neck

**Figure 4:**
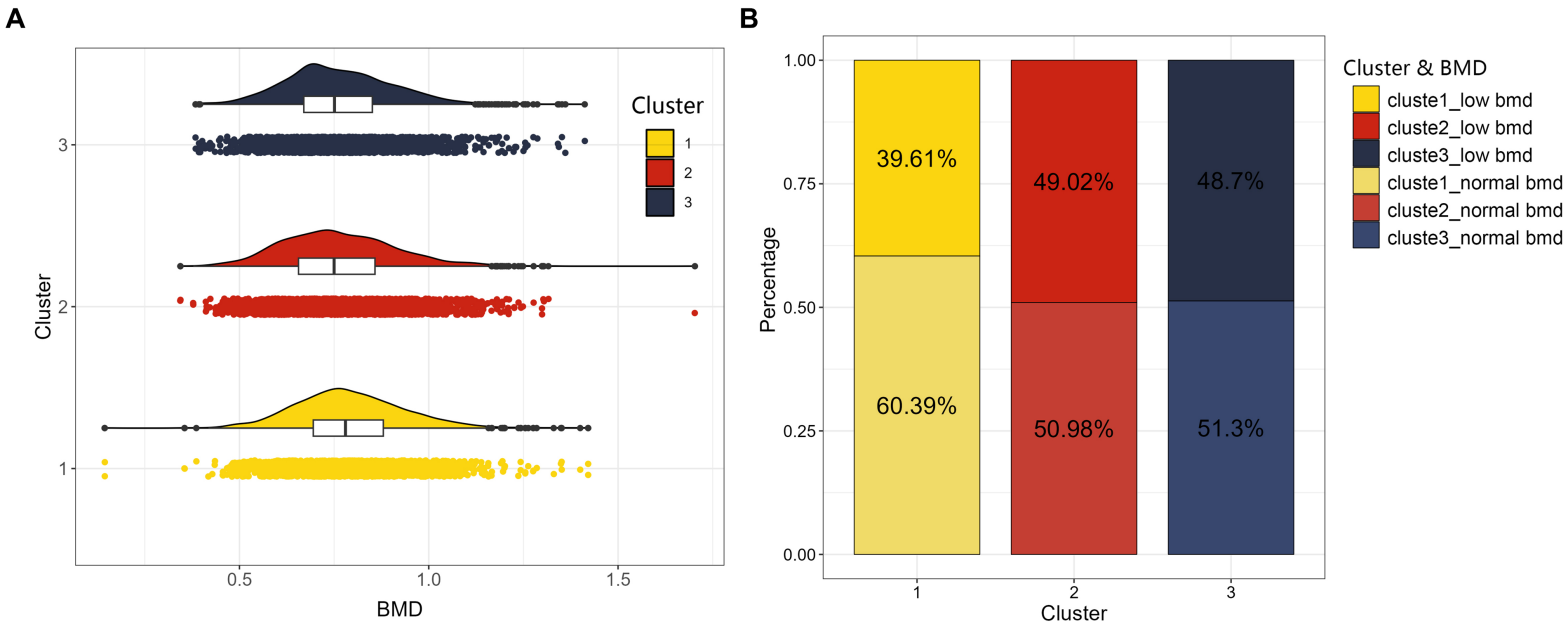
The distribution maps of three clusters. On the left **(A)** is cloud and rain plot of BMD as a continuous variable, and we can observe a bell-shaped distribution of BMD for the three clustered populations. On the right **(B)** is stacked bar chart of BMD converted into a categorical variable indicating whether individuals suffer from low BMD for the three clustered populations.

The populations of the three dietary patterns “LP-HDF-VitA-Mg,”"HM-Cho-Se” and “LM-VitD-Ca” consisted of 2,470, 2,570, and 3,276 individuals, respectively. Figure 4 displays the distribution of their femoral neck BMD and low BMD. Statistically significant differences were found in femoral neck BMD and low BMD among the three population patterns. The HM-Cho-Se pattern showed a slightly higher femoral neck BMD compared to the other two dietary patterns. Refer to Table 8 for specifics.

**Table 8:** Distribution of femoral neck BMD in the population with three dietary patterns.

Vars	Level	LP-HDF-VitA-Mg	HM-Cho-Se	LM-VitD-Ca	*χ*^2^/F	*p*
*N*		2,470	2,570	3,276		
BMD (mean (SD))		0.77 (0.14)	0.79 (0.14)	0.76 (0.15)	31.788	<0.001
Low BMD (%)	Normal	1,267 (51.3)	1,552 (60.4)	1,670 (51.0)	61.555	<0.001
	Low	1,203 (48.7)	1,018 (39.6)	1,606 (49.0)		

The relationship between dietary intake patterns and femoral neck BMD and risk of low BMD were shown in Table 9. In the correction model, the “HM-Cho-Se” pattern was positively correlated with BMD of femoral neck [*β* = 0.01 (−0.00,0.02), *p* = 0.025]. At the same time, compared with the “LP-HDF-VitA-Mg” mode, the risk of low BMD was 0.86 times [OR = 0.86 (0.75,0.99); *p* = 0.031], suggesting that better adherence to the “HM-Cho-Se” pattern may reduce the risk of low BMD. However, the “LM-VitD-Ca” pattern was not significantly associated with BMD in the femoral neck or the risk of developing low BMD.

**Table 9:** Association between dietary patterns and femoral neck BMD (corrected for demographic variables, BMI, smoke history and physical activity).

Vars	BMD of femur neck			low BMD		
	*β* [95% CI]	*t*	*p*	OR [95% CI]	*z*	*p*
(Intercept)	0.70 [0.67,0.74]	38.11	<0.001	2.87 [1.32,6.84]	2.538	0.011
Cluster [*LP-HDF-VitA-Mg*]						
HM-Cho-Se	0.01 [0.00,0.02]	2.25	0.025	0.86 [0.75,0.99]	−2.163	0.031
LM-VitD-Ca	0.01 [−0.00,0.01]	1.73	0.084	0.94 [0.83,1.07]	−0.885	0.376
Gender[Male]						
Female	−0.08 [−0.08,-0.07]	−24.86	<0.001	3.63 [3.22,4.10]	21.08	<0.001
Age group [40 ~ 55]						
55 ~ 70	−0.05 [−0.06,-0.05]	−14.99	<0.001	2.38 [2.06,2.76]	11.61	<0.001
≥ 70	−0.10 [−0.11,-0.09]	−23.29	<0.001	4.50 [3.79,5.34]	17.21	<0.001
Ethnicity [Mexican American]						
Other Hispanics	−0.00 [−0.02,0.02]	−0.06	0.954	1.02 [0.80,1.30]	0.16	0.877
Non-Hispanic White people	−0.02 [−0.03,-0.00]	−2.53	0.011	1.28 [1.05,1.56]	2.43	0.015
Non-Hispanic Black people	0.07 [0.06,0.09]	8.95	<0.001	0.42 [0.34,0.53]	−7.66	<0.001
Non-Hispanic Asians	−0.02 [−0.04,-0.00]	−2.46	0.014	1.56 [1.22,2.00]	3.54	<0.001
Other races	0.01 [−0.01,0.03]	1.17	0.242	0.95 [0.66,1.35]	−0.29	0.769
Education [Below high school]						
High school	−0.00[−0.01,0.01]	−0.84	0.403	0.96 [0.81,1.13]	−0.54	0.586
Above high school	−0.00[−0.01,0.00]	−1.34	0.180	1.08 [0.94,1.24]	1.08	0.281
Marital [Never married]						
Married/Living with partner	0.02 [0.01,0.03]	4.62	<0.001	0.80 [0.68,0.95]	−2.57	0.010
Widowed/Divorced/Separated	−0.00 [−0.02,0.01]	−0.86	0.387	1.02 [0.84,1.24]	0.22	0.826
Family income [low income]						
Middle income	0.01 [0.00,0.02]	1.96	0.050	0.83 [0.72,0.96]	−2.48	0.013
High income	0.02 [0.01,0.03]	4.32	<0.001	0.69 [0.60,0.80]	−5.01	<0.001
BMI[underweight]						
Normal	0.08 [0.05,0.11]	5.08	<0.001	0.18 [0.08,0.37]	−4.41	<0.001
Overweight	0.13 [0.10,0.16]	8.10	<0.001	0.09 [0.04,0.19]	−6.08	<0.001
Obesity	0.18 [0.15,0.21]	11.30	<0.001	0.05 [0.02,0.10]	−7.88	<0.001
Smoke[No]	0.00 [−0.00,0.01]	0.60	0.550	0.98 [0.87,1.10]	−0.34	0.733
Physical activity[high active]						
Insufficiently active	−0.01 [−0.02,-0.01]	−3.31	<0.001	1.23 [1.07,1.41]	2.86	0.004
Moderately active	−0.00 [−0.01,0.01]	−0.35	0.730	1.14 [0.98,1.32]	1.71	0.088

## 5 Discussion

In this study, women, higher age groups, and race were identified as risk factors for low bone mineral density (BMD). Numerous prior investigations have highlighted the significant influence of both gender and age on BMD. It was revealed that women are at a higher risk (OR = 3.33, 95% CI: 2.9–3.72, *p* < 0.001) of low BMD compared to men, while both the 55–70 age group (OR = 2.25, 95% CI: 1.96–2.59, *p* < 0.001) and the >70 age group (OR = 4.55, 95% CI: 3.86–5.36, *p* < 0.001) exhibited a higher risk of developing low BMD compared to the 40–55 age group. These findings corroborate earlier research indicating a progressive decline in BMD with advancing age, especially evident after 50 years, making middle-aged and elderly individuals more susceptible to osteoporosis. Additionally, existing literature has highlighted the rapid reduction in bone mass among women following menopause, attributable to declining estrogen levels. This phenomenon elucidates why middle-aged and elderly women exhibit a heightened vulnerability to osteoporosis compared to men of equivalent age (32).

Compared to Mexican Americans, Non-Hispanic White people have a higher risk of low BMD (OR = 1.28, 95% CI: 1.05–1.56, *p* = 0.015), as do Non-Hispanic Asians (OR = 1.56, 95% CI: 1.22–2.00, *p* < 0.001). Conversely, Non-Hispanic Black people have a lower risk of developing the condition (OR = 0.42, 95% CI: 0.34–0.53, *p* < 0.001). Previous studies have shown that, in general, Non-Hispanic Black adults tend to have the highest BMD and the lowest prevalence of bone loss compared to Hispanics, Mexican Americans, Non-Hispanic White people, and Non-Hispanic Asian adults (33, 34). The research findings indicate that polymorphism of the vitamin D receptor (VDR) may be associated with higher BMD in Non-Hispanic Black individuals compared to Non-Hispanic White individuals. Differences in VDR FokI genotypes suggest that allelic frequency differences between Non-Hispanic Black and Non-Hispanic White populations may contribute to BMD variation (35, 36). Additionally, genetic factors such as single nucleotide polymorphisms in TRPV6 and TRPV5 may result in more efficient calcium absorption in Non-Hispanic Black individuals (37, 38). Furthermore, cultural customs promoting physical activity and healthy lifestyles among Non-Hispanic Black populations may also contribute to their relatively higher BMD. In contrast, Non-Hispanic Asians tend to have lower BMD compared to other ethnicities, influenced by both genetic and lifestyle factors. This may be related to unfavorable VDR FokI and BsmI polymorphisms among Asians, which are associated with decreased BMD (39). Additionally, cultural factors such as dietary habits play a role in bone health. Traditional Asian diets typically include fewer dairy and calcium-rich foods, which may contribute to lower BMD (40).

This study also shows a positive correlation between BMI, physical activity, and bone mineral density (BMD). Both high BMI and high activity groups have a lower risk of low BMD, consistent with previous studies (41, 42). Firstly, mechanical loading on bones is greater with higher body weight (higher BMI) and more physical activity. This load generates stresses that activate osteoblasts, promoting bone formation and reducing bone resorption, thus increasing bone density. Physical activity also enhances bone health by increasing muscle strength and mass. Stronger muscles exert greater traction on bones during exercise, promoting bone growth and remodeling (43, 44). Secondly, hormonal regulation plays a role. Fat cells secrete hormones (e.g., estrogen), cytokines, and other signaling molecules that affect bone metabolism. Higher amounts of adipose tissue in obese (high BMI) individuals can alter these hormone levels, influencing bone density. Regular exercise increases levels of growth hormone and sex hormones (e.g., testosterone), crucial for bone metabolism. Additionally, BMI is an important indicator of nutritional status (45).

We extracted three dietary patterns, namely, the “LP-HDF-VitA-Mg” pattern, the “HM-Cho-Se” pattern, and the “LM-VitD-Ca” pattern, from the intake of 22 nutrients among 8,316 research subjects by combining factor analysis with cluster analysis. The proportions of the three dietary populations were 29.70, 30.91, and 39.39%, respectively, after clustering, with relatively balanced numbers of samples in the three categories. Significant differences in femoral neck densities were found between the groups (*F* = 31.788, *p* < 0.001). Factor analysis and cluster analysis are two post-hoc analysis methods commonly used in nutritional epidemiology to simplify data and reveal potential relationships between variables, helping to navigate the complex interrelationships among nutritional components and identify various dietary patterns. However, this process can be subject to considerable subjectivity in identifying common factors (46). Previous studies commonly divide factor scores into quartiles after extraction but erroneously assume that subjects with the highest scores represent the dietary pattern, leading to a loss of statistical information, as high scores in common factors merely indicate a tendency toward a specific dietary pattern (47). Cluster analysis complements factor analysis by dividing individuals into groups based on similarities in nutritional profiles, thus identifying subpopulations with similar dietary behaviors, providing a more nuanced view of nutritional patterns, and refining these patterns. In this study, factor analysis was used to identify dietary patterns based on participants’ nutrient intake factor scores, which were effectively categorized using cluster analysis to maximize the retention of original dietary information. Meanwhile, this study utilized the variance contribution ratio and scree plot for statistical analysis to capture the interconnections of nutrients and diverse dietary characteristics in the population.

Investigating the correlation between three dietary intake patterns and BMD, significant inter-group differences in BMD were initially found among the groups. Post-hoc pairwise comparisons revealed higher BMD in the “HM-Cho-Se” group compared to others. However, after adjusting for demographic variables, the difference in BMD between the “LM-VitD-Ca” pattern and the “LP-HDF-VitA-Mg” pattern group became nonsignificant. Yet, a positive correlation persisted with the “HM-Cho-Se” pattern, suggesting that it may mitigate the risk of low BMD.

Interesting findings from our study revealed a complex relationship between nutritional intake patterns and BMD. Higher BMD was observed in individuals who adhered to a dietary pattern rich in protein, carbohydrates, fats, choline, and selenium, emphasizing the potential synergistic effects of these nutrients on bone health. Protein is an essential component of the human diet and plays a key role in bone metabolism. Essential amino acids derived from dietary proteins are critical for the synthesis of collagen, a key structural protein in bone (48). The positive correlation between higher BMD and diets rich in carbohydrates and fats emphasizes the importance of energy availability in bone health. Carbohydrates serve as the body’s primary source of energy to fuel the metabolic processes required for optimal bone function. Similarly, dietary fat contributes to energy reserves and plays a role in hormone production, influencing bone metabolism (49). Choline and selenium, often overlooked in bone health discussions, emerged as potential contributors to higher BMD in our study. Although more research is needed to elucidate their specific mechanisms, choline is known to be involved in cell membrane synthesis, and selenium acts as an antioxidant that may protect bone tissue from oxidative stress (50, 51). The lack of significant differences in femoral BMD between individuals following a “LP-HDF-VitA-Mg” pattern and those adhering to a “LM-VitD-Ca” pattern suggests the need for further exploration. Differences in femoral BMD suggest that specific nutrients may interact in some way to counteract their individual effects on bone health in a given dietary pattern. This indicates the need for more in-depth research into the intricate interactions between nutrients and their combined effects on bone metabolism. At the same time, we need to recognize that individual differences, lifestyle factors, and physical activity levels are critical to the potential impact of study results. Individuals respond differently to various nutritional patterns, which, together with external factors such as exercise and lifestyle choices, may lead to variations in the relationship between diet and bone health.

Our study has several limitations. Firstly, since the NHANES femoral scans were conducted only on individuals aged 40 and above, our findings are not generalizable to a wider age range. Skeletal health is influenced by various factors throughout life, and limiting our sample to those over 40 may miss BMD changes in younger populations. Secondly, using femoral BMD as the sole outcome indicator diminishes the reliability of our results. While femoral BMD is an important measure of bone health, it does not fully represent overall skeletal health and may be affected by individual differences, measurement accuracy, and method consistency. Relying on a single metric may overlook other important aspects of bone health, limiting the study’s applicability and accuracy. Additionally, this study’s cross-sectional design makes it difficult to establish a causal relationship between nutritional patterns and BMD. A longitudinal study would better capture the temporal aspects of these associations and assess the impact of dietary changes on bone health over time. Lastly, factor analysis introduces potential subjectivity, affecting the reliability of identified dietary patterns and their relationship with BMD. Future studies should include diverse age groups, use a longitudinal design, adopt comprehensive covariates, and employ more objective data analysis measures to address these limitations.

## 6 Conclusion

In summary, our study shows that individuals adhering to a dietary regimen characterized by elevated levels of macronutrients, choline, and selenium demonstrate a diminished likelihood of experiencing low BMD. This suggests that the consumption of foods rich in these specific nutrients may confer benefits for skeletal health.

## Data Availability

Publicly available datasets were analyzed in this study. This data can be found at: https://wwwn.cdc.gov/nchs/nhanes/Default.aspx.
